# Pre-Operative Sonographic Assessment of Ovarian Location and Mobility Predicts Intra-Operative Ovarian Resectability During Vaginal Hysterectomy: A Diagnostic Accuracy Study

**DOI:** 10.3390/diagnostics16060952

**Published:** 2026-03-23

**Authors:** Iakovos Theodoulidis, Nikolaos Roussos, Menelaos Zafrakas, Christos Anthoulakis, Pantelis Trompoukis, Grigorios F. Grimbizis, Themistoklis Mikos

**Affiliations:** 1 1st Department of Obstetrics & Gynecology, Aristotle University of Thessaloniki, Papageorgiou General Hospital, 56403 Thessaloniki, Greece; jakobtheod@hotmail.com (I.T.); nikosroussos1992@hotmail.com (N.R.); christos.anthoulakis@hotmail.com (C.A.); pantelistrompoukis@gmail.com (P.T.); grigoris.grimbizis@gmail.com (G.F.G.); 2 School of Health Science, International Hellenic University, 57400 Thessaloniki, Greece

**Keywords:** ultrasound, vaginal surgery, ovarian mobility, salpingo-oophorectomy, adhesions, diagnostic study

## Abstract

**Background/Objectives**: This study investigates the predictive role of pre-operative sonographic assessment of ovarian mobility in determining intra-operative ovarian resectability among patients undergoing vaginal hysterectomy for pelvic organ prolapse. **Methods**: This prospective study was conducted in a tertiary academic urogynecology center. Women with pelvic organ prolapse scheduled for vaginal hysterectomy were consecutively recruited after providing informed consent. Pre-operatively, all patients had a detailed history, pelvic examination (POP-Q), and pelvic floor ultrasound (including assessment of the mobility of both ovaries and sonographic determination of ovarian descent in relation to the pelvic ischial spines). Patients were planned for vaginal hysterectomy, anterior and posterior colporrhaphy, McCall culdoplasty, and bilateral salpingo-oophorectomy (SO), where feasible. During surgery, the location and mobility of the ovaries, as well as the presence of peri-ovarian adhesions, were recorded. Pair-to-pair comparisons between sonographic and clinical findings were performed. **Results**: From February 2023 to January 2024, 50 Caucasian adult women underwent reconstructive vaginal surgery for prolapse. Thirty-five patients underwent concomitant bilateral SO via vaginal route, seven underwent unilateral SO, and three under went salpingectomy only. ROC analysis indicated that pre-operative ultrasound assessment of ovarian mobility predicts: (1) intra-operative ovarian mobility (sensitivity 95.6%, specificity 77.8%); (2) the presence of peri-ovarian adhesions (sensitivity 46.1%, specificity 94.2%); and (3) resectability, i.e., the ability to perform SO via the vaginal route (sensitivity 96.4%, specificity 50.0%). The absence of ovarian mobility was not associated with an increased risk of intra-operative and post-operative complications. **Conclusions**: Pre-operative sonographic assessment of ovarian location and mobility can predict ovarian location and resectability during vaginal surgery with high diagnostic accuracy.

## 1. Introduction

Hysterectomy with or without bilateral oophorectomy is the most common procedure for benign uterine disease. It can be performed abdominally, vaginally, laparoscopically or robotically [1]. Vaginal hysterectomy is performed as the gold standard procedure for the treatment of pelvic organ prolapse, providing great relief to women experiencing symptoms such as pressure and pain via a minimally invasive technique [2]. Vaginal hysterectomy provides the advantage of shorter recovery and hospital stay, with decrease demands for post-operative analgesia [3].

However, the choice of removing the adnexa at the time of vaginal hysterectomy can be affected by many variables, such as ovarian mobility, difficulties in transvaginal surgical accessibility in the higher pelvis, fear of complications, and inadequate surgical training [4,5]. Kovac et al. proposed a grading system to assess the difficulty of removing the ovaries vaginally based on the possibility of pulling them down from their anatomical position. They concluded that ovaries that can be mobilized at the level of the ischial spines can be removed via the transvaginal route [6]. Therefore, understanding and analyzing ovarian mobility before surgery is crucial for achieving a successful and complication-free outcome. The sonographic assessment of the location and mobility of the ovaries has been well described in the literature; thus, pre-operative investigation of these features in the setting of reconstructive surgery could assist in higher surgical precision and pre-operative selection of the appropriate instruments or techniques in order to facilitate the surgical approach [7,8]. To our knowledge, there has been no reports comparing the sonographic evaluation of ovarian location and mobility with clinical findings during vaginal hysterectomy.

In this study, our aim was to investigate the predictive role of pre-operative sonographic assessment of ovarian mobility in determining intra-operative ovarian mobility and resectability among patients undergoing vaginal hysterectomy for pelvic organ prolapse.

## 2. Materials and Methods

This prospective study was conducted in a tertiary academic urogynecology center between February 2023 and January 2024, according to the Standards for Reporting of Diagnostic Accuracy Studies (STARD) (see Supplementary Table S1). Ethical committee approval was obtained (Papageorgiou General Hospital, Thessaloniki, Scientific and Ethics Committee Approval, Decision Number 2021-Β2015-131). Women with pelvic organ prolapse (POP) scheduled for reconstructive surgery were consecutively recruited into the study after providing informed consent. The following inclusion criteria were used: patients with POP with involvement of the mid compartment (uterine prolapse grade 2 or more) and patients’ wish not to preserve the uterus during POP surgery. The exclusion criteria were as follows: history of removal of the ovaries, previous hysterectomy, and history of chemotherapy or pelvic radiation.

Demographics including the age, height, weight, and Body Mass Index (BMI) of the women, the Last Menstrual Period (LMP), the number of years in menopause, as well as gravidity, parity, mode of delivery, and birth weight of the heaviest new born were obtained.

Pre-operatively, all patients had a pelvic examination with evaluation of POP using the POP-Q (IUGA/ICS, Pelvic Organ Prolapse Quantification system)and a transvaginal scan (TVS). TVS included assessment of the mobility of both ovaries and sonographic determination of ovarian descent in relation to the pelvic ischial spines [7]. All sonographic examinations were performed by the same physician (I.T.) using an imaging system (Voluson S10 BT16; GE Healthcare) equipped with a transvaginal probe (5–9 MHz). At the outset of the study, the operator (I.T.) was a specialist in Obstetrics and Gynecology with more than five years of experience in pelvic floor ultrasound. The ultrasound operator was not blinded to clinical information. According to the study design, sonographic data were recorded separately for each ovary rather than each patient, taking into account that ovarian mobility and the surrounding and/or adherent tissues may differ considerably within the same woman, and that some patients may have only one ovary or one adnexum due to previous operations.

Patients were planned for vaginal hysterectomy, anterior and posterior colporrhaphy, McCall culdoplasty, and bilateral salpingo-oophorectopy (BSO) where feasible. All surgeries were performed by the same team of pelvic floor surgeons. The following peri- and post-operative data were recorded: type of anesthesia; descent of the ovaries in relation to the ischial spines and the hymen; presence of pelvic adhesions; intra-operative complications; operative time; uterine volume; and use of analgesia on the first and second post-operative days. All data was stored and analyzed in Microsoft Excel. Thus, a direct pair-to-pair comparison between the pre-operative sonographic findings and the intra-operative findings regarding the location of the ovaries, the mobility of the ovaries, and the presence of peri-adnexal adhesions was feasible for each participant.

### 2.1. Sonographic Assessment of Ovarian Mobility: Description of the Technique

Sonographic examinations were performed transvaginally in the lithotomy position with an empty urinary bladder. The length of the ovaries was obtained in an oblique sagittal section and their width and height in the frontal section after a 90° rotation of the transducer. The volume of the ovaries was estimated using the formula for the volume of an ellipsoid (0.523 × longitudinal diameter × transverse diameter × anteroposterior diameter). The mobility of the ovaries was assessed using the real-time TVS pelvic sliding sign, as described by Hudelist et al. [8]. The examiner applied gentle pressure with the TVS probe on each ovary in order to determine whether the organ glided freely in relation to the adjacent organs. To confirm sliding, the structure could be further mobilized by applying abdominal pressure with the examiner’s free hand on the left or right lower abdomen. Specifically, a positive sliding sign was defined when the area of interest (adnexum) slid freely after applying pressure with the transvaginal probe or after applying abdominal pressure. If no sliding was observed, this was defined as a negative sliding sign [9]. The mobility of the ovaries was described as high, moderate, or absent. Ovaries that did not present any movement were characterized as “‘not mobile”. Ovaries that presented movement with gentle transvaginal or abdominal pressure were characterized as “fully mobile”. Ovaries that presented movement only after increased abdominal pressure were characterized as “moderately mobile” [9,10,11].

### 2.2. Sonographic Evaluation of Ovarian Location: Description of the Technique

The sonographic evaluation of the location of the ovary was based on the proximity of the ovary to certain unanimously accepted anatomic pelvic landmarks: ovaries close to the internal iliac vessels were described as higher located, whereas ovaries close to the corpus uteri or the uterine cervix were described as lower located [12].

### 2.3. Intra-Operative Evaluation of Ovarian Descent: Description of the Technique

The surgeon was not aware of the sonography results. The peri-operative findings were contingent upon visualization of adhesions during surgery. Mobility of the ovaries was assessed intra-operatively using a long Allis clamp inserted transvaginally; the ovary was described as mobile if it was possible to rotate the ovary and expose the lateral pelvic wall.

The position/descent of the ovaries in the surgical field was described in relation to the level of ischial spines and the upper vagina in the same way as described in a system used to classify the clinical grading of descent in POP: “Grade 0” prolapse described immobile ovaries; “Grade 1” prolapse described ovaries with descent between their normal position and the ischial spines; “Grade 2” prolapse described ovaries with descent between the ischial spines and the hymen; “Grade 3” prolapse described ovaries with descent reaching the hymen; and “Grade 4” prolapse described ovaries with descent further beyond the hymen [13]. In the current study, the following modification was used: “Grade 0”—no descent; the infundibulopelvic ligament has little or no stretchability; “Grade I”—the ovaries can be retracted halfway between the ischial spines and the midportion of the vagina; “Grade II”—the ovaries can be retracted between the midvagina and the hymenal ring; and “Grade III”—the ovaries can be retracted past the hymenal ring [6]. For statistical analysis, cases with ovaries having Grade 0 mobility were classified as “ovaries with no mobility”, whereas cases with the ovaries having Grade I, II or III mobility were classified as “mobile” ovaries.

### 2.4. Statistical Analysis

Data were analyzed to define the accuracy of the pre-operative TVS sliding sign in predicting ovarian mobility and the presence of pelvic adhesions confirmed during vaginal surgery. Continuous variables were presented as mean values with 95% confidence intervald (C.I.). The sensitivity, specificity, positive predictive value (PPV) and negative (NPV) predictive value, as well as the positive (LR+) and negative (LR-) likelihood ratios, were calculated for the TVS sliding sign in this setting. Receiver-operating characteristics (ROC) curve analysis was performed where possible. The chi-square test was used to test the statistical significance of the prediction of ovarian movement and pelvic adhesions using the sliding sign. *p* < 0.05 was considered statistically significant. Given the possibility of ovarian non-mobility to be 15%, and in order to obtain an area under the curve (AUC) of 0.800 (Type I error rate 0.05 and 1-βerror 0.8), a sample of 63 cases (32 women) was calculated as an adequately powered study group. The statistical software package Comprehensive Meta-Analysis (CMA), Version 3.3.070, 2014 (Biostat Inc., 14 North Dean Street, Englewood, NJ 07631, USA) and Medcalc for Windows, version 12.7 (Medcalc Software, Mariakerke, Belgium) were used for data analyses.

## 3. Results

**Figure 1:**
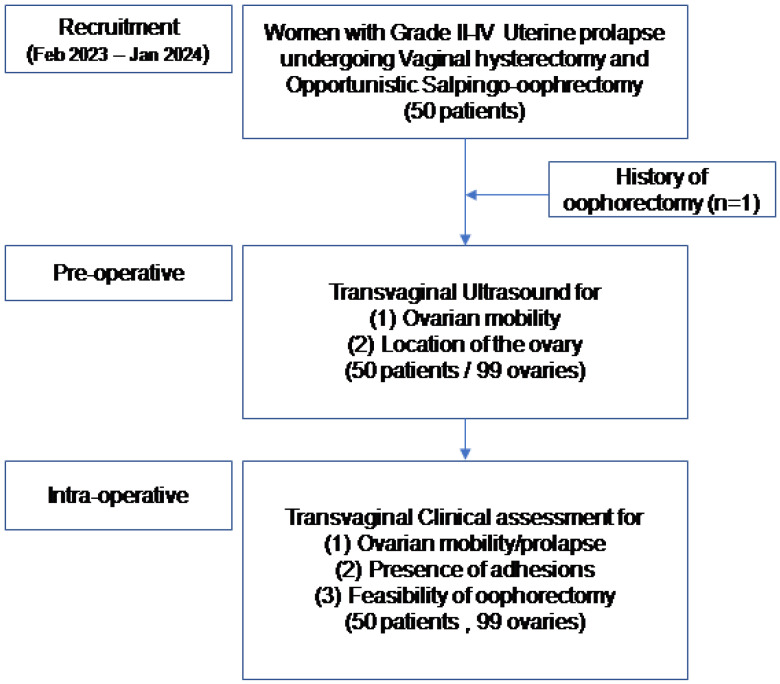
Study flowchart.

During this 12-month study, a total of 50 Caucasian adult women were consecutively enrolled. Their mean age was 66.0 (±8.8) years old, mean BMI was 28.8 (±4.2) kg/m^2^, and mean parity was 2.2 (±0.5) births. There was a case with a history of unilateral salpingo-oophorectomy (1/100, 1%). All demographics and clinical pre-operative details are shown in Table 1. The study flowchart is presented in Figure 1.

All patients had pelvic organ prolapse and underwent vaginal hysterectomy± transvaginal BSO, culdoplasty, and anterior or posterior repair when appropriately, according to the local protocol. Thirty-five patients underwent concomitant bilateral salpingo-oophorectomy (BSO) via the vaginal route, seven patients underwent unilateral salpingo-oophorectomy, and three patients underwent only bilateral salpingectomy (two of them were of reproductive age). BSO was not performed in five patients, in two cases due to their pre-operative wish and in the remaining three cases due to difficulty during the trial of transvaginal BSO and subsequent abandoning of the procedure. Epidural and spinal anesthesia were given to 90% of patients, and general anesthesia was given to 10% of patients. The mean time for performing a vaginal hysterectomy with unilateral salpingo-oophorectomy was 126 (±25.6) min, whereas the mean time when only vaginal hysterectomy was performed was119 (±15.0) min. Interestingly, the absence of ovarian motility was not associated with an increased risk of intra-operative and post-operative complications.

Pre-operative sonographic evaluation of the mobility and location of the ovaries indicated that 88 out of 99 (88.9%) ovaries had moderate or good mobility and that 64 out of 99 (64.7%) ovaries were observed in close proximity to the internal iliac vessels compared to 35 out of 99 (35.3%) observed closer to the uterus. During surgery, 90 out of 99 (90.1%) ovaries had moderate or good mobility; 64 out of 99 (64.7%) ovaries were found lying below or at the level of the ischial spines compared to 35 out of 99 (35.3%) ovaries found above the level of the ischial spine; adhesions were encountered in 13 out of 99 (13.1%) tubo-ovarian areas, and salpingo-oophorectomy or salpingectomy was performed in 83 out of 99 ovaries (83.8%) (Table 2).

Ultrasound assessment of ovarian location correctly identified the place of the ovaries in 95 out of 99 (96.0% accuracy) cases (sensitivity = 97%, specificity = 94%). The sonographic assessment of ovarian location was related to the presence or absence of peri-adnexal adhesions in 71 out of 99 (71.7% accuracy) cases (sensitivity = 76.9%, specificity = 70.9%) and to successful salpingo-oophorectomy or simple oophorectomy in 74 out of 99 (74.7% accuracy) cases (sensitivity = 73.5%, specificity = 81.2%) (Table 3).

Ultrasound assessment of ovarian mobility using the positive sliding sign correctly identified intra-operative ovarian mobility in 93 out of 99 (93.4% accuracy) cases (sensitivity = 95.6%, specificity = 77.8%). The sonographic assessment of ovarian mobility was related to the presence or absence of peri-adnexal adhesions in 86 out of 99 (87.9% accuracy) cases (sensitivity = 46.2%, specificity = 94.2%) and to the feasibility of salpingo-oophorectomy or simple oophorectomy in 88 out of 99 (88.9% accuracy) cases (sensitivity = 96.4%, specificity = 50.0%) (Table 4).

**Figure 2:**
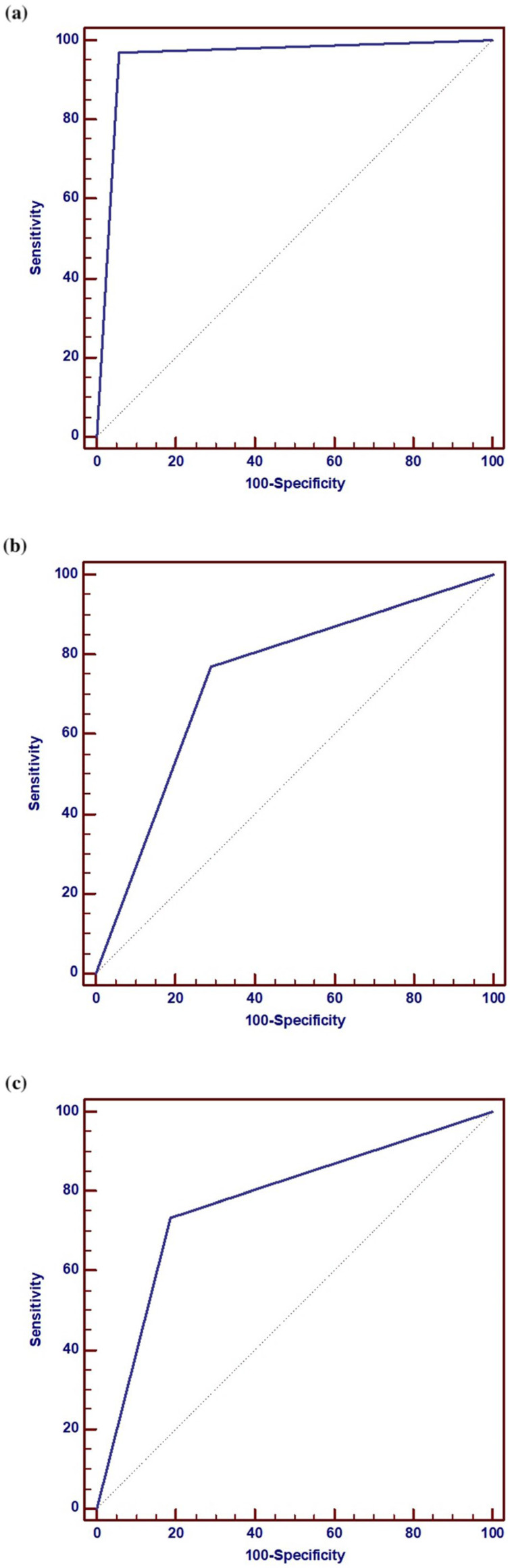

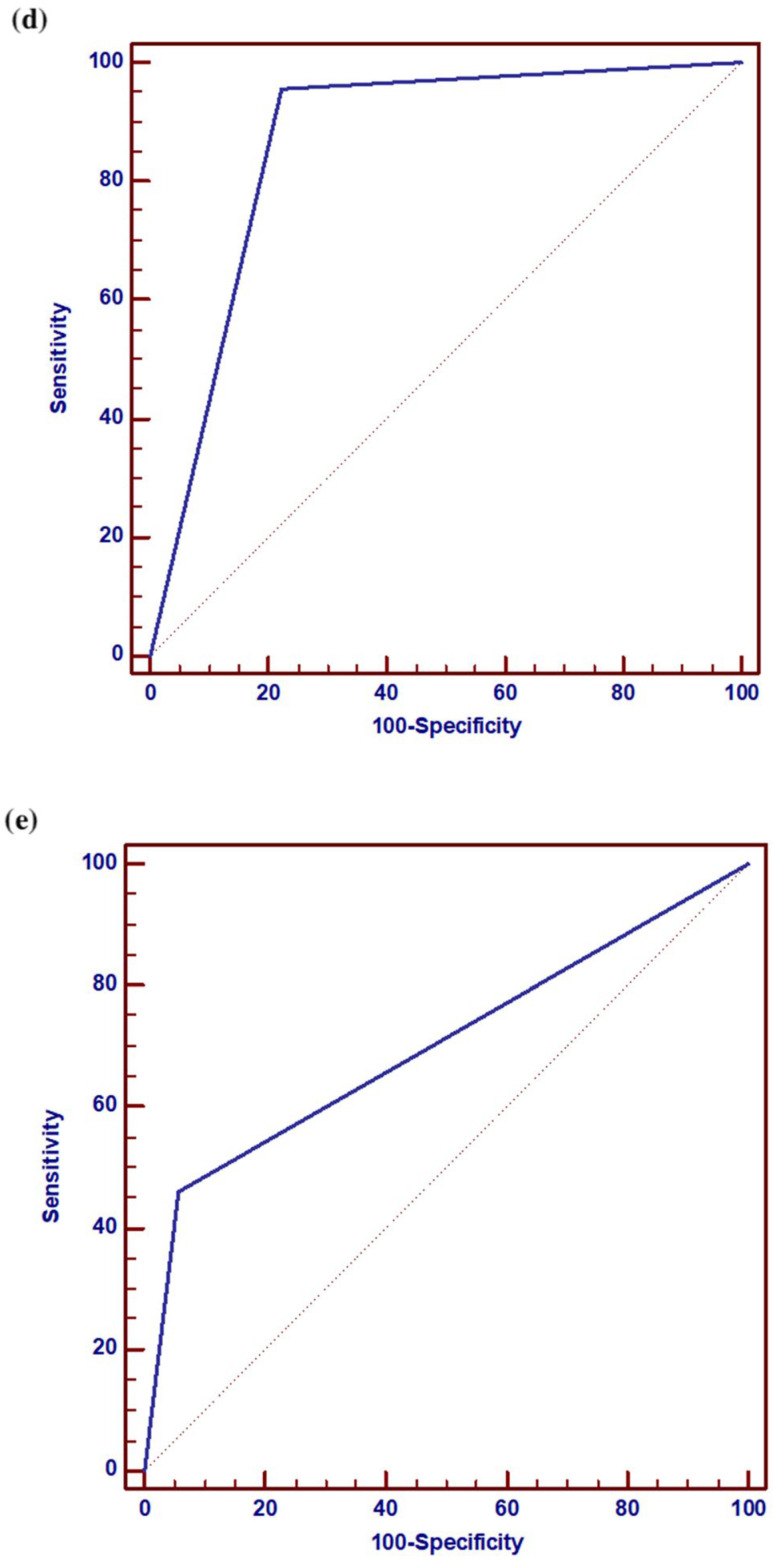

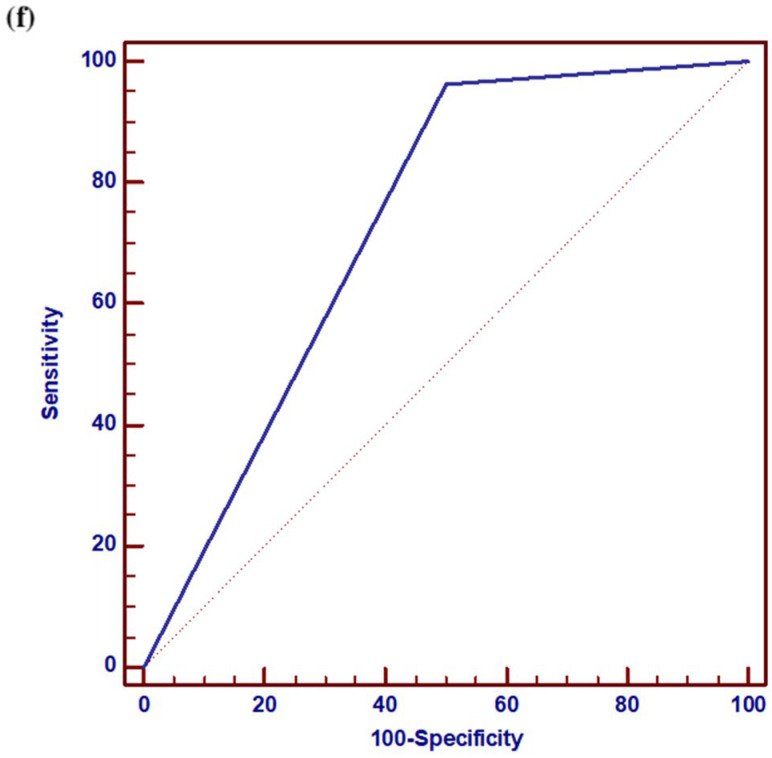
ROC curves. (**a**) Diagnostic performance of the use of sonographic assessment of ovarian location for the intra-operative location of the ovary. (**b**) Diagnostic performance of the use of sonographic assessment of ovarian location for the intra-operative presence of peri-ovarian adhesions. (**c**) Diagnostic performance of the use of sonographic assessment of ovarian location for the feasibility of vaginal BSO during vaginal hysterectomy. (**d**) Diagnostic performance of the use of sonographic assessment of ovarian mobility for the intra-operative mobility of the ovary. (**e**) Diagnostic performance of the use of sonographic assessment of ovarian mobility for the intra-operative presence of peri-ovarian adhesions. (**f**) Diagnostic performance of the use of sonographic assessment of ovarian mobility for the feasibility of vaginal BSO during vaginal hysterectomy.

ROC analysis indicated that the area under the curve (AUC) of the sonographic assessment of ovarian location in relation to the location of the ovary during surgery, the presence of adhesions, and the feasibility of vaginal BSO during vaginal hysterectomy was 0.956, 0.739, and 0.774, respectively. Similarly, the AUC for the sonographic assessment of ovarian mobility in relation to ovarian mobility during surgery, the presence of adhesions, and the feasibility of vaginal BSO during vaginal hysterectomy was 0.867, 0.702, and 0.732, respectively (Table 5). It appears that the sonographic assessment of ovarian location and mobility prior to transvaginal surgery using the sliding test is highly predictive of the place of the ovary during surgery, the presence of adhesions, and the feasibility of transvaginal BSO. ROC curves for all comparisons are presented in the Figure 2.

## 4. Discussion

The aim of this study was to investigate the feasibility of pre-operative sonographic assessment of ovarian mobility in a group of peri- and post-menopausal women with pelvic organ prolapse scheduled for vaginal reconstructive surgery. Our results indicate that the pre-operative sonographic assessment of ovarian mobility can be predictive of ovarian mobility during vaginal hysterectomy, with very good sensitivity, specificity and accuracy.

Our results demonstrate an excellent correlation between the pre-operative sonographic assessment and the intra-operative confirmation of ovarian location and mobility (AUC 0.956 and 0.867, respectively). Today, given the multitude of surgical options for intra-operative access to the adnexa (transvaginal route, use of v-NOTES, use of conventional laparoscopy), this information is essential in order to design, prepare in advance, and meet the surgical demands of each case. For example, the pre-operative finding of mobile ovaries located close to the uterine corpus indicates increased feasibility of inspection and/or removal. In contrast, the pre-operative finding of non-mobile ovaries or ovaries close to the internal iliac vessels indicate an increased possibility of using an endoscopic approach, such as v-NOTES or laparoscopy, in order to examine and/or remove them.

The sliding test and the evaluation of ovarian mobility are not new methods, as they have been extensively studied in the field of endometriosis, specifically in the diagnostic approach to deep infiltrating endometriosis [10]. In a recent systematic review and meta-analysis, the researchers included 936 patients and concluded that, when performed by an expert, the sliding test has a sensitivity of 88% (95% CI, 81–93%) and a specificity of 94% (95% CI, 91–96%) for detecting pouch of Douglas obliteration and bowel involvement [14]. Young et al. proposed that the sliding test can be part of the routine ultrasound examination of women who present with chronic pelvic pain or have a history of endometriosis [15]. Consistently, Piessenset al. suggested that the sliding test can be part of routine ultrasound examination in the investigation of deep infiltrating endometriosis [16]. The novelty of our study is the application of this knowledge in the field of reconstructive urogynecology, challenging the notion that the widespread use of ultrasound lacks significant clinical value in this area. Moreover, this is the one of the first studies comparing pre-operative sonographic ovarian mobility with intra-operative findings in the setting of transvaginal surgery.

Again, using the knowledge from studies dealing with chronic pelvic pathology, pelvic adhesions are well known causes of chronic pelvic pain, dyspareunia, and correlate with higher difficulty performing surgery [11,17]. In terms of assessing pelvic adhesions with ultrasound, there are a few studies in the literature, and most of them have focused on utilizing transabdominal (not transvaginal) ultrasound in the estimation of pelvic adhesions. Ayachi et al. [9] were the first to use transvaginal ultrasound for the pre-operative estimation of pelvic adhesions in women with a history of surgery in the abdomen. These researchers concluded that transvaginal ultrasound is more predictive for adhesions concerning the uterine fundus and the adnexa [9]. Thus, the presence of a negative sliding test can be suggestive of adhesions or pelvic inflammation. Pre-operative knowledge of adnexa adhesions is important information for the gynecologist who plans to perform transvaginal BSO, leading to the decision to start the operation laparoscopically or to be prepared for alternative surgical set-ups such as v-NOTES. However, using the transvaginal probe is more difficult for imaging adhesions higher in the pelvis. In such cases, transabdominal ultrasound can be beneficial [18].

The literature lacks solid proof about the proficiency of the examiner performing pre-operative ultrasound. According to Tammaaet al., the learning curve requires a sufficient number of cases to depict reliability [19], while Leonardi et al. proposed that not all trainees can reach proficiency in diagnosing bowel endometriosis in a predetermined number of scans [20]. This parameter may hamper the generalization of our results, as well as the availability of transvaginal ultrasound in pelvic floor disease centers and surgical clinics.

This study has multiple strengths. The prospective, blind (in that the surgeon was not aware of the sonography results), and adequately powered study design corroborates our results. Performance of all ultrasound examinations by a single, experienced operator, and strict criteria for the sonographic and intra-operative diagnosis of ovarian mobility and the place of the ovaries, construct a robust methodology. Moreover, the use of ROC curves sends a clear message that ultrasound is a tool that, with novel indications, may further improve everyday gynecology practice.

One limitation of our study is that all ultrasound examinations were performed by a single examiner, precluding inter-observer variability analysis. Although this limits generalizability, it improves the consistency of the findings. Likewise, our prospective study design did not include intra-observer variability analysis. Yet these limitations are counterbalanced by existing relevant knowledge from studies performed in the setting of deep endometriosis and chronic pelvic pain. Another possible limitation is that the physician who performed the ultrasound examinations was not blinded to clinical information, and this may have altered diagnosis; however, in real life, outside the frame of a research study, the physician would have to be aware of patients’ clinical information. A practical limitation may be the fact that ultrasound is still not routinely used in the urogynecology setting, and thus the generalizability of the results may be restricted from the fact that many units do not possess such modalities in their practice. Finally, our study group includes only Caucasian women, so cross-racial implementation of these findings may be inappropriate.

## 5. Conclusions

In conclusion, the pre-operative sonographic assessment of ovarian location and mobility can be predictive of ovarian location, mobility and resectability during vaginal surgery, with high diagnostic accuracy. Yet, interpretation of our findings should be cautious, as prediction of some factors with pre-operative ultrasound, such as the presence of adhesions, is not as accurate as the prediction of ovarian mobility and resectability. The pre-operative assessment of ovarian mobility by a non-invasive and well-tolerated technique could assist in better planning the surgery, improving the quality of care provision to the patient. Further studies involving a higher number of participants from different ethnic background are warranted in order to confirm these findings and clarify whether and how pre-operative ultrasound examination assessing ovarian mobility could be incorporated into practical clinical algorithms in this setting.

## Figures and Tables

**Table 1:** Baseline characteristics, demographics and ultrasound assessments.

Baseline Characteristics		Statistics
Total number of patients (N)		50
Age (years, Mean, SD)		66.02 (8.80)
BMI (kg/m^2^, Mean, SD)		28.17(4.24)
Parity (N, SD)		2.24 (0.52)
BWHN, (SD) g		3662 (425.65)
Race/ethnicity (%)		Caucasian, 100%
Years in menopause (years, Mean, SD)		19.50 (11.01)
POP-Q: GH (cm, Mean, SD)		3.58 (0.56)
POP-Q: C (cm, Mean, SD)		1.50 (2.42)
POP-Q: D (cm, Mean, SD)		−2.24(2.10)
**Ultrasound Measurements**		
Uterine volume (cm^3^, Mean, SD)		24.04(19.40)
Left ovary volume(cm^3^, Mean, SD)		4.91 (6.54)
Right ovary volume(cm^3^, Mean, SD)		3.90 (6.70)
Descent of right ovary ^1^	Proximal to the int.iliac vessels	20/49 (40.82%)
	Proximal to the uterus	29/49 (59.18%)
Descent of left ovary	Proximal to the int.iliac vessels	15/50 (30.00%)
	Proximal to the uterus	35/50 (70.00%)
Right ovary mobility ^1^	High	36/49 (73.47%)
	Moderate	9/49 (18.37%)
	No	4/49 (8.16%)
Left ovary mobility	High	38/50 (76.00%)
	Moderate	5/50 (10.00%)
	No	7/50 (14.00%)

^1^ One patient had a history of unilateral salpingo-oophorectomy. SD: standard deviation, BMI: Body Mass Index, BWHN: body weight of heavier newborn, POP-Q: pelvic organ prolapse quantification.

**Table 2:** Intra-operative and post-operative results.

Intra-Operative Results			Statistics
Total number of patients, N			50
Surgical time (min, Mean, SD)		Total	126.94 (25.67)
		Bilateral salpingo-oophorectomy (n = 35)	126.00 (25.65)
		Unilateral salpingo-oophorectomy (n = 7)	123.00 (29.26)
		No salpingo-oophorectomy (n = 5)	119.17(15.00)
		Only salpingectomy (n = 3)	161.00 (16.82)
Type of anesthesia		Epidural and Spinal	45/50 (90%)
		General	5/50 (10%)
Descent of right ovary ^1^		Above the level of ischial spine	19/49 (38.78%)
		Below or at the level of ischial spine	30/49 (61.22%)
Descent of left ovary		Above the level of ischial spine	16/50 (32.00%)
		Below or at the level of ischial spine	34/50 (68.00%)
Right ovary mobility ^1^		High	37/49 (75.51%)
		Moderate	7/49 (14.29%)
		No	5/49 (10.20%)
Left ovary mobility		High	40/50 (80.00%)
		Moderate	6/50 (12.00%)
		No	4/50 (8.00%)
Pelvic adhesions			7/50 (14.00%)
Intra-operative complications			0/50 (0.00%)
**Post-operative Results**			
Post-operative complications			0/50 (0.00%)
Use of analgesia	First post-operative day		46/50 (92.00%)
	Second post-operative day		0/50 (0.00%)

^1^ One patient had a history of unilateral salpingo-oophorectomy.

**Table 3:** Ultrasound pre-operative assessment of ovarian location as a prediction tool.

Prediction	Sensitivity	Specificity	Accuracy	+LR	−LR	+PV	−PV
Ovarian location	96.87	94.29	95.96	16.95	0.033	96.9	94.3
Presence of peri-ovarian adhesions	76.92	70.93	71.72	2.65	0.33	28.6	95.3
Feasibility of TV SO	73.49	81.25	74.75	3.92	0.33	95.3	37.1

TV SO = Transvaginal salpingo-oophorectomy; LR = likelihood ratio; PV = predictive value.

**Table 4:** Ultrasound pre-operative ovarian mobility as a prediction tool.

Prediction	Sensitivity	Specificity	Accuracy	+LR	−LR	+PV	−PV
Intra-operative ovarian mobility	95.56	77.78	93.94	4.30	0.057	97.7	63.6
Presence of peri-ovarian adhesions	46.15	94.19	87.88	7.94	0.57	54.5	92.0
Feasibility of TV SO	96.39	50.00	88.89	1.93	0.072	90.9	72.7

TV SO = transvaginal salpingo-oophorectomy; LR = likelihood ratio; PV = predictive value.

**Table 5:** Calculation of diagnostic accuracy of sonographic assessment of adnexa location and mobility.

Comparison	AUC	C.I.
US location of adnexa vs. Sx location of adnexa	0.956	0.895 to 0.987
II. US location of adnexa vs. Sx adhesions	0.739	0.641 to 0.822
III. US location of adnexa vs. oophorectomy	0.774	0.679 to 0.852
IV. US adnexa mobility vs. Sx adnexa mobility	0.867	0.874 to 0.927
V. US adnexa mobility vs. Sx adhesions	0.702	0.601 to 0.790
VI. US adnexa mobility vs. oophorectomy	0.732	0.633 to 0.816

AUC = area under the curve; C.I. = confidence interval; US = sonographic; Sx = intra-operative.

## Data Availability

The original contributions presented in this study are included in the article. Further inquiries can be directed to the corresponding authors.

## Supplementary Materials

The following supporting information can be downloaded at: https://www.mdpi.com/article/10.3390/diagnostics16060952/s1. Table S1: STARD checklist.

## Abbreviations

The following abbreviations are used in this manuscript:

AUC	Area under the curve
BMI	Body mass index
BSO	Bilateral salpingo-oophorectomy
BWHN	Body weight of heavier newborn,
CI	Confidence interval
ICS	International Continence Society
IUGA	International Uragynecology Association
LMP	Last menstrual period
LR	Likelihood ratio
NPV	Negative predictive value
POP	Pelvic organ prolapse
POP-Q	Pelvic organ prolapse quantification system
PPV	Positive predictive value
ROC	Receiver-operating characteristics
SD	Standard deviation
TVS	Transvaginal scan
