# Supplementary material for: Pre-Operative Sonographic Assessment of Ovarian Location and Mobility Predicts Intra-Operative Ovarian Resectability During Vaginal Hysterectomy: A Diagnostic Accuracy Study
Source: Diagnostics (Basel). 2026 Mar 23;16(6):952. doi: 10.3390/diagnostics16060952 (PMC13024837; doi:10.3390/diagnostics16060952)
Supplement: Supplementary file 1 [file diagnostics-16-00952-s001.zip › diagnostics-4182902-supplementary.pdf]

Supplementary Materials::

1. Table S1: STARD checklist.

Section & Topic	No	Item	Reported on page #
**TITLE OR ABSTRACT**			
	**1**	Identification as a study of diagnostic accuracy using at least one measure of accuracy (such as sensitivity, specificity, predictive values, or AUC)	#1.
**ABSTRACT**			
	**2**	Structured summary of study design, methods, results, and conclusions (for specific guidance, see STARD for Abstracts)	#3,4
**INTRODUCTION**			
	**3**	Scientific and clinical background, including the intended use and clinical role of the index test	#5
	**4**	Study objectives and hypotheses	#6
**METHODS**			
Study design	**5**	Whether data collection was planned before the index test and reference standard were performed (prospective study) or after (retrospective study)	#7
Participants	**6**	Eligibility criteria	#7
	**7**	On what basis potentially eligible participants were identified (such as symptoms, results from previous tests, inclusion in registry)	#7
	**8**	Where and when potentially eligible participants were identified (setting, location and dates)	#7
	**9**	Whether participants formed a consecutive, random or convenience series	#7
Test methods	**10a**	Index test, in sufficient detail to allow replication	#
	**10b**	Reference standard, in sufficient detail to allow replication	#8-9
	**11**	Rationale for choosing the reference standard (if alternatives exist)	#8-9
	**12a**	Definition of and rationale for test positivity cut-offs or result categories of the index test, distinguishing pre-specified from exploratory	#8-9
	**12b**	Definition of and rationale for test positivity cut-offs or result categories of the reference standard, distinguishing pre-specified from exploratory	#8-9
	**13a**	Whether clinical information and reference standard results were available to the performers/readers of the index test	#8-9
	**13b**	Whether clinical information and index test results were available to the assessors of the reference standard	#8-9
Analysis	**14**	Methods for estimating or comparing measures of diagnostic accuracy	#10
	**15**	How indeterminate index test or reference standard results were handled	#10
	**16**	How missing data on the index test and reference standard were handled	#10
	**17**	Any analyses of variability in diagnostic accuracy, distinguishing pre-specified from exploratory	#10
	**18**	Intended sample size and how it was determined	#10

**RESULTS**			
Participants	**19**	Flow of participants, using a diagram	Figure 1
	**20**	Baseline demographic and clinical characteristics of participants	#11
	**21a**	Distribution of severity of disease in those with the target condition	#11
	**21b**	Distribution ofalternative diagnoses in those without the target condition	#11
	**22**	Time interval and any clinical interventions between index test and reference standard	#11
Test results	**23**	Cross tabulation of the index test results (or their distribution) by the results of the reference standard	#11-12, Tables 3-5
	**24**	Estimates of diagnostic accuracy and their precision (such as 95% confidence intervals)	#11-12, Tables 3-5
	**25**	Any adverse events from performing the index test or the reference standard	#11-12, Tables 3-5
**DISCUSSION**			
	**26**	Study limitations, including sources of potential bias, statistical uncertainty, and generalisability	#15-16
	**27**	Implications for practice, including the intended use and clinical role of the index test	#13-14
**OTHER INFORMATION**			
	**28**	Registration number and name of registry	#7
	**29**	Where the full study protocol can be accessed	On demand
	**30**	Sources of funding and other support; role of funders	No external funding
